# Comparison of long-term cardiovascular and renal outcomes between percutaneous coronary intervention and coronary artery bypass grafting in multi-vessel disease with chronic kidney disease

**DOI:** 10.3389/fcvm.2022.951113

**Published:** 2022-09-12

**Authors:** Woochan Kwon, Ki Hong Choi, Dong Seop Jeong, Sang Yoon Lee, Joo Myung Lee, Taek Kyu Park, Jeong Hoon Yang, Joo-Yong Hahn, Seung-Hyuk Choi, Su Ryeun Chung, Yang Hyun Cho, Kiick Sung, Wook Sung Kim, Hyeon-Cheol Gwon, Young Tak Lee, Young Bin Song

**Affiliations:** ^1^Division of Cardiology, Department of Internal Medicine, Samsung Medical Center, Sungkyunkwan University School of Medicine, Heart Vascular Stroke Institute, Seoul, South Korea; ^2^Department of Thoracic and Cardiovascular Surgery, Samsung Medical Center, Sungkyunkwan University School of Medicine, Heart Vascular Stroke Institute, Seoul, South Korea

**Keywords:** coronary artery disease, percutaneous coronary intervention, coronary artery bypass grafting, chronic renal insufficiency, clinical outcomes

## Abstract

**Objective:**

This study aims to analyze cardiac and renal outcomes of chronic kidney disease (CKD) patients with multi-vessel disease who have undergone coronary artery bypass grafting (CABG) or percutaneous coronary intervention (PCI).

**Materials and methods:**

Chronic kidney disease patients with multi-vessel disease who underwent CABG or PCI were retrospectively selected from our database and divided into the PCI group [further stratified into PCI with complete revascularization (PCI-CR) and PCI with incomplete revascularization (PCI-IR) groups] and the CABG group. The primary endpoint was the composite of all-cause death, myocardial infarction (MI), or stroke at 5 years. The key secondary endpoint was the 5-year rate of the renal composite outcome, defined as >40% glomerular filtration rate decrease, initiation of dialysis, and/or kidney transplant. Outcomes were compared using Cox proportional hazards regression analysis, and the results were further adjusted by multivariable analyses and inverse probability weighting.

**Results:**

Among the study population (*n* = 798), 443 (55.5%) patients received CABG and 355 (44.5%) patients received PCI. Compared with the CABG group, the PCI group had similar risk of the primary endpoint (CABG vs. PCI, 19.3% vs. 24.0%, HR: 1.28, 95% CI: 0.95–1.73, *p* = 0.11) and a lower risk of the renal composite outcome (36.6% vs. 31.2%, HR: 0.74, 95% CI 0.58–0.94, *p* = 0.03). In addition, PCI-IR was associated with a significantly higher risk of the primary endpoint than CABG (HR: 1.54, 95% CI: 1.11–2.13, *p* = 0.009) or PCI-CR (HR: 1.78, 95% CI: 1.09–2.89, *p* = 0.02). However, PCI-CR had a comparable 5-year death, MI, or stroke rate to CABG (HR: 0.86, 95% CI 0.54–1.38, *p* = 0.54).

**Conclusion:**

Coronary artery bypass grafting showed an incidence of death, MI, or stroke similar to PCI but was associated with a higher risk of renal injury. PCI-CR had a prognosis comparable with that of CABG, while PCI-IR had worse prognosis. If PCI is chosen for revascularization in patients with CKD, achieving CR should be attempted to ensure favorable outcomes.

**Clinical trial registration:**

[clinicaltrials.gov], identifier [NCT 03870815].

## Introduction

Chronic kidney disease (CKD) is an important risk factor for coronary artery disease, associated not only with its development and progression but also with morbidity and mortality thereafter (1). Patients with CKD alone have a higher mortality risk after myocardial infarction (MI) than those with diabetes mellitus or a prior history of MI (2). Several studies have reported that coronary artery bypass grafting (CABG), compared with percutaneous coronary intervention (PCI), is associated with a higher risk of short-term death and stroke but reduced long-term risk of MI or repeat revascularization in patients with CKD (3, 4). Patients with CKD are also at risk for acute kidney injury (AKI), a common complication after CABG or PCI (5), and for progression to end-stage renal disease (6). Although many studies have concluded that PCI preserves renal function better (5, 7), most of these studies have been conducted on a short-term follow-up, and data on other kidney-related outcomes such as initiation of dialysis or kidney transplant are scarce.

Outcomes of PCI can differ according to completeness of revascularization. Previous observational studies have found significant benefit of complete revascularization (CR) compared with incomplete revascularization (IR) (8, 9). Outcomes of PCI with CR can be similar to those of CABG (10). Cardiac and renal outcomes have not been comprehensively compared between PCI and CABG, especially according to completeness of revascularization of PCI. We therefore compared long-term cardiovascular and renal outcomes among patients with CKD and multi-vessel disease who received CABG or PCI based on the data from a comprehensive institutional registry.

## Materials and methods

### Study population and data collection

**FIGURE 1:**
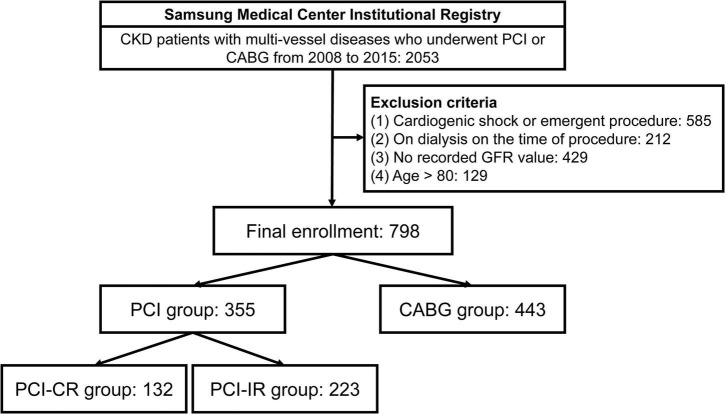
Study flow. CKD, chronic kidney disease; PCI, percutaneous coronary intervention; CABG, coronary artery bypass graft; GFR, glomerular filtration rate; PCI-CR, percutaneous coronary intervention with complete revascularization; PCI-IR, percutaneous coronary intervention with incomplete revascularization.

This was a retrospective, single-center, and observational study conducted from January 2008 to December 2015. During this time period, CKD patients with multi-vessel disease (defined as having two or more diseased coronary arteries) who underwent CABG or PCI were recruited. The data were taken from a comprehensive registry of Samsung Medical Center consisting of adult (older than 18 years) patients with ischemic heart disease who underwent CABG or PCI with second-generation drug-eluting stents (clinicaltrials.gov, NCT 03870815). Exclusion criteria for this study were cardiogenic shock, emergent procedure, age over 80 years, being on dialysis before the procedure, and unavailable glomerular filtration rate (GFR) data. Finally, 798 subjects were enrolled and divided into the PCI group (*n* = 355) and CABG group (*n* = 443). The PCI group was further stratified into PCI-CR (*n* = 132) and PCI-IR (*n* = 223) groups (Figure 1).

Baseline demographic, angiographic, and procedural data, and adverse outcomes during the follow-up period were collected prospectively from our registry by research coordinators. Additional information was obtained from medical records and telephone interviews, if necessary. Mortality data for patients lost to follow-up were confirmed from the national death records. All events were adjudicated by a cardiologist who was blinded to the treatment strategy. The study protocol was approved and requirement for informed consent was waived by the Institutional Review Board of Samsung Medical Center. This study was conducted in accordance with the Declaration of Helsinki.

All revascularization procedures are described in detail in Supplementary material.

### Definitions and outcomes

Chronic kidney disease was defined as a GFR less than 60 mL/min/1.73 m^2^ as calculated by using the CKD Epidemiology Collaboration equation, both at the baseline and post-procedure stabilization (11). Multi-vessel disease was defined as the presence of luminal stenosis 50% or greater in two or more major epicardial arteries on coronary angiography. In the PCI group, anatomic CR was defined as visually estimated residual stenosis < 30% in vessels with a diameter of 2.25 mm or wider coronary branches with Thrombolysis In Myocardial Infarction grade 3 flow in all arteries at the end of the procedure (12); all other cases were defined as anatomic IR.

The primary endpoint was major adverse cardiac and cerebrovascular events (MACCE), a composite of all-cause death, MI, or stroke at 5 years. The key secondary endpoint was the 5-year rate of renal composite outcomes, defined as a GFR decrease of more than 40% of the baseline value, new requirement for dialysis (both emergent and elective), or kidney transplant. Other secondary endpoints included all-cause death, cardiac death, MI, stroke, and repeat revascularization at 5 years. All deaths were considered cardiac-related, unless a definite non-cardiac cause was established. MI was defined as elevated cardiac enzymes (troponin or myocardial band fraction of creatine kinase) greater than the upper reference limit with concomitant ischemic symptoms or electrocardiography findings indicative of ischemia. Periprocedural MI was not included as a clinical event. Stroke was defined as a neurological deficit attributable to an acute focal injury of the central nervous system caused by cerebral infarction or hemorrhage, with the supporting findings on brain imaging. Repeat revascularization was defined as unplanned revascularization for any coronary arteries after the index PCI or CABG procedure.

### Statistical analysis

Categorical variables are presented as numbers and relative frequencies (%) and were compared using the chi-square test. Continuous variables are presented as means ± standard deviations or median with interquartile range according to distribution of data as tested by the Kolmogorov–Smirnov test, and were analyzed using Welch’s *t*-test or Mann–Whitney U test, respectively. The cumulative incidence of primary and secondary endpoints at 5 years were calculated using the Kaplan–Meier method and were compared between groups using the log-rank test. Cardiovascular and renal outcomes were compared using Cox proportional hazard regression models with calculation of hazard ratios (HRs) and 95% confidence intervals (CIs). Multivariable models for Cox regression included age, sex, acute MI at presentation, hypertension, diabetes mellitus, left ventricular ejection fraction < 40%, history of stroke, presence of left main involvement, and multi-vessel disease. Inverse probability-weighted (IPW) analyses were also performed. The probability was calculated using twang function on statistics software R (R version 4.0.5, R Foundation for Statistical Computing, Vienna, Austria) with various factors, a full list of which is provided in Supplementary Table 1 with their respective standardized mean differences before and after IPW adjustments. IPW-adjusted Cox proportional hazard models were used to compare outcomes between the groups. A landmark analysis was performed to compare short-term and long-term MACCE and renal composite outcomes between the two groups.

All probability values were two-sided, and *P*-values < 0.05 were considered statistically significant. Statistical analyses were performed using SPSS version 25 for Windows (SPSS-PC, Chicago, IL, United States) and R. All tests were two-tailed, and *p* < 0.05 was considered statistically significant.

## Results

### Baseline characteristics

The mean age and estimated GFR of the total study population were 68.7 ± 7.3 years and 45.9 ± 12.5 mL/min/1.73 m^2^, respectively. Baseline clinical characteristics according to the treatment strategy are shown in Table 1. Patients who underwent CABG were younger and more likely to have a history of stroke than those who underwent PCI. On the contrary, patients who underwent PCI were more likely to have a history of coronary revascularization and diabetes mellitus. Left main involvement and three-vessel disease were more frequent in the CABG group than in the PCI group.

**TABLE 1:** Comparison of baseline characteristics according to the revascularization method.

Variables	CABG (*N* = 443)	PCI (*N* = 355)	*P*-value
**Demographics**			
Age (years)	70.0 [64.0, 74.0]	71.0 [65.0, 75.0]	0.005
Male	303 (68.4%)	249 (70.1%)	0.60
Body mass index (kg/m^2^)	24.6 [22.7, 26.8]	24.6 [22.8, 26.5]	0.92
**Cardiovascular risk factors**			
Hypertension	352 (79.5%)	280 (78.9%)	0.84
Diabetes mellitus	293 (66.1%)	258 (72.7%)	0.05
History of myocardial infarction	43 (9.7%)	46 (13.0%)	0.15
History of PCI	83 (18.7%)	88 (24.8%)	0.04
History of CABG	7 (1.6%)	31 (8.7%)	<0.001
History of stroke	83 (18.7%)	41 (11.5%)	0.01
**Initial presentation**			
Diagnosis at presentation			
Acute myocardial infarction	62 (14.0%)	60 (16.9%)	0.26
Stable angina	209 (47.2%)	225 (63.4%)	<0.001
Unstable angina	172 (38.8%)	70 (19.7%)	<0.001
Pre-procedure LVEF (%)	56.4 [42.5, 64.0]	59.0 [46.0, 65.0]	0.03
LVEF < 40% at baseline	105 (23.7%)	125 (35.2%)	<0.001
Creatinine (mg/dL)	1.4 [1.2, 1.7]	1.4 [1.2, 1.7]	0.99
Glomerular filtration rate (mL/min/1.73 m^2^)	49.5 [38.8, 55.9]	50.8 [38.0, 55.7]	0.86
Hemoglobin (mg/dL)	11.9 [10.5, 13.3]	12.0 [11.0, 14.0]	0.001
C-reactive protein (mg/L)	0.2 [0.1, 0.8]	0.2 [0.1, 0.5]	0.01
**Coronary anatomy**			
Left main involvement	79 (17.8%)	27 (7.6%)	<0.001
Number of involved vessels			<0.001
2-vessel disease	93 (21.0%)	186 (52.4%)	
3-vessel disease	350 (79.0%)	169 (47.6%)	
**Medication at discharge**			
Aspirin	430 (97.1%)	353 (99.4%)	0.03
P2Y12 inhibitor	322 (72.7%)	353 (99.4%)	<0.001
Beta blocker	372 (84.0%)	220 (62.0%)	<0.001
RAS blocker	121 (27.3%)	264 (74.4%)	<0.001
Statin	317 (71.6%)	330 (83.0%)	<0.001

Values are means ± standard deviations (or median [1^st^ interquartile, 3^rd^ interquartile]) or numbers (%). CABG, coronary artery bypass graft; PCI, percutaneous coronary intervention; LVEF, left ventricular ejection fraction; RAS, renin–angiotensin system.

Baseline characteristics of the two PCI groups and the CABG group are presented in Supplementary Table 2, and the procedural characteristics of the PCI groups and the CABG group are summarized in Supplementary Tables 3, 4, respectively. Compared with the PCI-CR group, the PCI-IR group was more likely to have acute MI at presentation, three-vessel disease, and history of previous MI and/or coronary revascularization. The lesion number and location were similar between the two PCI groups, but chronic total occlusion was more frequent in the PCI-IR group than in the PCI-CR group. A trans-radial approach was more frequently used with higher volume of contrast in the PCI-CR group than in the PCI-IR group. The mean implanted stent number was significantly higher in the PCI-CR group than in the PCI-IR group.

### Comparison of outcomes between the coronary artery bypass grafting and percutaneous coronary intervention groups

**FIGURE 2:**
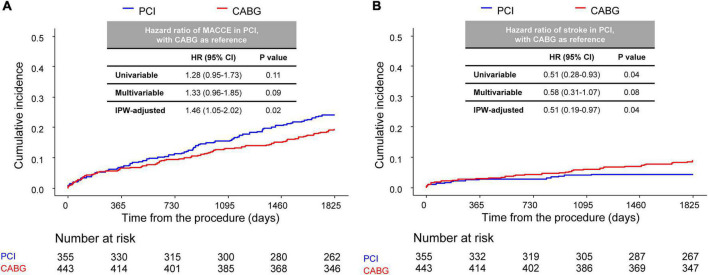
Comparison of 5-year MACCE between CABG and PCI. Kaplan–Meier curve comparing **(A)** MACCE and **(B)** stroke at the 5-year point between the CABG and PCI groups. CABG, coronary artery bypass graft; PCI, percutaneous coronary intervention; MACCE, major adverse cardiac and cerebrovascular events.

The median follow-up duration of the cohort was 2.77 (interquartile range 0.97–5.27) years. Compared with CABG, PCI had a similar risk of MACCE at 5 years (PCI vs. CABG, 24.0% vs. 19.3%, HR 1.28, 95% CI 0.95–1.73, *p* = 0.11) (Figure 2A and Table 2), although the risk was significantly higher in the PCI group after IPW adjustment. The risk of repeat revascularization was significantly higher in the PCI group than in the CABG group (PCI vs. CABG, 11.1% vs. 2.2%, HR 5.35, 95% CI 2.58–11.10, *p* < 0.001). By contrast, patients treated with PCI had a significantly lower risk of stroke (PCI vs. CABG, 4.4% vs. 8.8%, HR 0.51, 95% CI 0.28–0.93, *p* = 0.04) and renal composite outcome (PCI vs. CABG, 31.2% vs. 36.6%, HR 0.74, 95% CI 0.58–0.94, *p* = 0.03) than those treated with CABG (Figure 2B and Table 2). The results were mostly consistent after adjustment for baseline differences in multivariable and IPW analyses.

**TABLE 2:** Comparison of clinical outcomes at 5 years in the CABG and PCI groups.

Variables	CABG (*N* = 443)	PCI (*N* = 355)	Univariable analysis		Multivariable analysis*		IPW analysis	
			HR (95% CI)	*P*-value	HR (95% CI)	*P*-value	HR (95% CI)	*P*-value
MACCE	85 (19.3%)	85 (24.0%)	1.28 (0.95–1.73)	0.11	1.33 (0.96–1.85)	0.09	1.46 (1.05–2.02)	0.02
Death	57 (13.0%)	71 (20.0%)	1.59 (1.13–2.26)	0.01	1.61 (1.10–2.35)	0.01	1.63 (1.13–2.35)	0.01
Cardiac death	36 (8.4%)	42 (12.3%)	1.49 (0.96–2.33)	0.08	1.47 (0.90–2.38)	0.12	1.52 (0.95–2.42)	0.08
Acute myocardial infarction	4 (1.0%)	10 (3.1%)	3.24 (1.02–10.33)	0.05	3.92 (1.14–13.45)	0.03	2.99 (0.92–9.75)	0.07
Stroke	37 (8.8%)	15 (4.4%)	0.51 (0.28–0.93)	0.04	0.58 (0.31–1.07)	0.08	0.51 (0.19–0.97)	0.04
Repeat revascularization	9 (2.2%)	36 (11.1%)	5.35 (2.58–11.10)	<0.001	5.99 (2.78–12.88)	<0.001	5.10 (2.42–10.8)	<0.001
Renal composite outcome†	160 (36.6%)	107 (31.2%)	0.74 (0.58–0.94)	0.03	0.71 (0.54–0.93)	0.01	0.77 (0.59–0.99)	0.05

*Multivariable adjusted analysis was performed with the variables of age over 70 years, sex, acute myocardial infarction at presentation, hypertension, diabetes mellitus, left ventricular ejection fraction under 40%, history of stroke, left main coronary artery involvement, and three-vessel disease.

^†^Renal composite outcome was defined as a decrease in the GFR of more than 40%, initiation of dialysis, or kidney transplant during the follow-up.

All analyses were performed with the CABG group as the reference.

CABG, coronary artery bypass graft; PCI, percutaneous coronary intervention; IPW, inverse probability weighting; HR, hazard ratio; CI, confidence interval; MACCE, major cardiac and cerebrovascular events.

**FIGURE 3:**
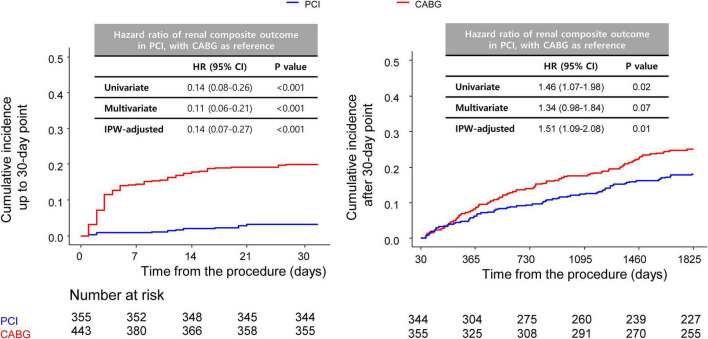
Comparison of 5-year renal outcomes between CABG and PCI. Kaplan–Meier curve comparing 5-year renal composite outcomes between the PCI and CABG groups with landmark analysis before **(left)** and after **(right)** 30 days. CABG, coronary artery bypass graft; PCI, percutaneous coronary intervention.

A landmark analysis was performed to assess the risk of PCI or CABG on both short-term and long-term follow-ups. A similar incidence of MACCE of the PCI and CABG groups was shown both before and after 1-year point, although CABG showed a slight trend toward a lower incidence of MACCE compared with the PCI group (Supplementary Figure 1). Meanwhile, compared with CABG, PCI had beneficial effects on the risk of the renal composite outcome within 30 days of the index procedure, with continued divergence of the curves throughout the study period (Figure 3).

### Comparison of outcomes among the percutaneous coronary intervention with complete revascularization, percutaneous coronary intervention with incomplete revascularization, and coronary artery bypass grafting groups

**FIGURE 4:**
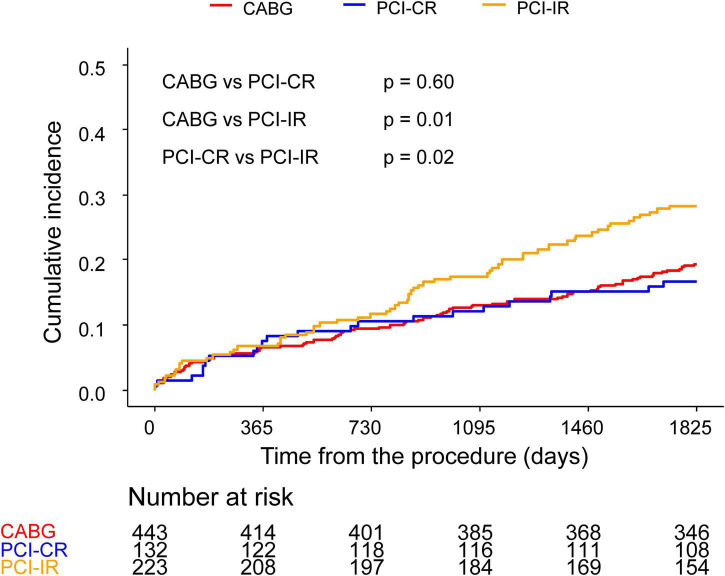
Comparison of 5-year MACCE among the CABG, PCI-CR, and PCI-IR groups. Kaplan–Meier curves comparing MACCE at 5 years among the CABG and two PCI groups. CABG, coronary artery bypass graft; PCI, percutaneous coronary intervention; MACCE, major adverse cardiac and cerebrovascular events.

There was a significant difference in the incidence of 5-year MACCE between patients who were treated with PCI-IR and those treated with CABG (PCI-IR vs. CABG, 28.3% vs. 19.3%, HR 1.54, 95% CI 1.11–2.13, *p* = 0.009) or PCI-CR (PCI-IR vs. PCI-CR, 28.3% vs. 16.7%, HR 1.78, 95% CI 1.09–2.89, *p* = 0.02), but there was no significant difference in the incidence of 5-year MACCE between patients treated with PCI-CR and those treated with CABG (PCI-CR vs. CABG, 16.7% vs. 19.3%, HR 0.86, 95% CI 0.54–1.38, *p* = 0.54) (Figure 4 and Tables 3, 4). Compared with patients treated with CABG, those treated with PCI-CR showed comparable cardiac death, MI, and stroke rates. However, there was a significantly higher incidence of repeat revascularization in the PCI-CR group in addition to the PCI-IR group than in the CABG group (Tables 3, 4 and Supplementary Figure 2). Meanwhile, there were fewer adverse renal outcomes in the PCI-CR group than in the CABG group (renal composite outcome in the PCI-CR vs. CABG groups, 26.9% vs. 36.6%, HR 0.64, 95% CI 0.44–0.92, *p* = 0.02), while the incidence of the renal composite outcome in the PCI-IR group was not significantly different from that of the CABG or PCI-CR groups (Tables 3, 4).

**TABLE 3:** Cumulative incidence of events at 5 years in the CABG, PCI-CR, and PCI-IR groups.

Variables	CABG (*N* = 443)	PCI-CR (*N* = 132)	PCI-IR (*N* = 223)	*P*-value (CABG vs PCI-CR)	*P*-value (CABG vs PCI-IR)	*P*-value (PCI-CR vs PCI-IR)
MACCE	85 (19.3%)	22 (16.7%)	63 (28.3%)	0.60	0.01	0.02
Death	57 (13.0%)	17 (12.9%)	54 (24.3%)	1.00	<0.001	0.02
Cardiac death	36 (8.4%)	9 (7.0%)	33 (15.6%)	0.76	0.01	0.04
Acute myocardial infarction	4 (1.0%)	2 (1.6%)	8 (4.0%)	0.91	0.03	0.42
Stroke	37 (8.8%)	6 (4.6%)	9 (4.4%)	0.20	0.06	1.00
Repeat revascularization	9 (2.2%)	10 (8.1%)	26 (12.9%)	0.004	<0.001	0.30
Renal composite outcome*	160 (36.6%)	35 (26.9%)	72 (33.8%)	0.05	0.37	0.31

*Renal composite outcome was defined as a decrease in the GFR of more than 40%, initiation of dialysis, or kidney transplant during the follow-up.

CABG, coronary artery bypass graft; PCI-CR, percutaneous coronary intervention with complete revascularization; PCI-IR, percutaneous coronary intervention with incomplete revascularization; MACCE, major cardiac and cerebrovascular events.

**TABLE 4:** Clinical outcomes at 5 years in the CABG, PCI-CR, and PCI-IR groups.

Variables	Univariable analysis		Multivariable analysis*		IPW analysis	
	HR (95% CI)	*P*-value	HR (95% CI)	*P*-value	HR (95% CI)	*P*-value
***CABG vs. PCI-CR (CABG as reference)***						
MACCE	0.86 (0.54–1.38)	0.54	0.88 (0.53–1.49)	0.64	0.88 (0.53–1.48)	0.63
Death	0.99 (0.57–1.69)	0.96	0.99 (0.54–1.82)	0.98	1.06 (0.58–1.92)	0.86
Cardiac death	0.83 (0.40–1.71)	0.61	0.81 (0.36–1.82)	0.62	0.86 (0.38–1.93)	0.72
Acute myocardial infarction	1.66 (0.30–9.07)	0.55	1.15 (0.16–8.01)	0.89	1.41 (0.25–7.81)	0.39
Stroke	0.54 (0.23–1.27)	0.16	0.64 (0.25–1.66)	0.36	0.53 (0.21–1.35)	0.18
Repeat revascularization	3.78 (1.54–9.30)	0.004	3.27 (1.17–9.10)	0.02	3.94 (1.54–10.00)	0.004
Renal composite outcome^†^	0.64 (0.44–0.92)	0.02	0.60 (0.40–0.90)	0.01	0.64 (0.44–0.94)	0.02
***CABG vs. PCI-IR (CABG as reference)***						
MACCE	1.54 (1.11–2.13)	0.009	1.59 (1.12–2.27)	0.01	1.68 (1.21–2.04)	0.002
Death	1.98 (1.36–2.87)	<0.001	1.94 (1.30–2.89)	0.001	2.18 (1.49–3.19)	<0.001
Cardiac death	1.91 (1.19–3.07)	0.01	1.80 (1.08–3.00)	0.02	2.16 (1.34–3.49)	0.002
Acute myocardial infarction	4.27 (1.28–14.17)	0.02	6.59 (1.77–24.58)	0.005	4.56 (1.35–15.40)	0.02
Stroke	0.50 (0.24–1.03)	0.06	0.58 (0.27–1.26)	0.17	0.50 (0.24–1.04)	0.07
Repeat revascularization	6.33 (2.96–13.51)	<0.001	6.97 (3.15–15.45)	<0.001	6.21 (2.89–13.3)	<0.001
Renal composite outcome^†^	0.80 (0.61–1.06)	0.13	0.74 (0.55–1.00)	0.05	0.90 (0.68–1.19)	0.45
***PCI-CR vs. PCI-IR (PCI-CR as reference)***						
MACCE	1.78 (1.09–2.89)	0.02	1.71 (1.04–2.82)	0.03	2.09 (1.21–3.60)	0.008
Death	2.01 (1.17–3.47)	0.01	1.93 (1.11–3.36)	0.02	2.06 (1.13–3.77)	0.02
Cardiac death	2.32 (1.11–4.84)	0.03	2.24 (1.06–4.74)	0.04	2.50 (1.11–5.65)	0.03
Acute myocardial infarction	2.49 (0.53–11.71)	0.25	2.20 (0.44–10.92)	0.34	3.15 (0.65–15.3)	0.16
Stroke	0.91 (0.32–2.54)	0.85	0.78 (0.27–2.26)	0.65	0.92 (0.30–2.77)	0.88
Repeat revascularization	1.68 (0.81–3.49)	0.16	1.56 (0.74–3.27)	0.24	1.60 (0.75–3.43)	0.23
Renal composite outcome^†^	1.29 (0.86–1.93)	0.22	1.20 (0.79–1.81)	0.39	1.44 (0.94–2.21)	0.10

*Multivariable adjusted analysis was performed with the variables of age over 70 years, sex, acute myocardial infarction at presentation, hypertension, diabetes mellitus, left ventricular ejection fraction under 40%, history of stroke, left main coronary artery involvement, and three-vessel disease.

^†^Renal composite outcome was defined as a decrease in the GFR of more than 40%, initiation of dialysis, or kidney transplant during the follow-up.

CABG, coronary artery bypass graft; PCI-CR, percutaneous coronary intervention with complete revascularization; PCI-IR, percutaneous coronary intervention with incomplete revascularization; IPW, inverse probability weighting; HR, hazard ratio; CI, confidence interval; MACCE, major cardiac and cerebrovascular events.

## Discussion

We evaluated the impact of different revascularization methods on cardiovascular and renal outcomes in patients with CKD. We also assessed whether outcomes of PCI depend on the completeness of revascularization. Our major findings are as follows. First, PCI is associated with a risk of MACCE similar to CABG but protects kidney function better than CABG in patients with CKD. Second, PCI-CR has a risk of MACCE at 5 years comparable to CABG. Third, PCI-IR is associated with a higher risk of MACCE than both PCI-CR and CABG.

### Cardiovascular adverse events

Several studies have investigated cardiovascular outcomes after PCI or CABG in patients with CKD. While CABG was reported to be associated with a higher risk of short-term mortality and stroke, MI and repeat revascularization were less frequent than in those who had undergone PCI in the long-term follow-up (3, 4, 13, 14). The majority of these studies designated MACCE as the primary outcome and concluded CABG is associated with fewer adverse events in patients with CKD than in PCI (14, 15). However, some studies have published contrary findings (16, 17), most likely due to differences in the stent type and definitions of the primary outcome. Therefore, we sought to better clarify the risk of PCI in the current-generation drug-eluting stenting and compare it with that of CABG. We found a similar incidence of the composite of all-cause death, MI, or stroke at 5 years in the PCI groups, even after thorough adjustment of baseline differences. In addition, secondary cardiovascular outcomes were largely in concordance with previous studies. The incidence of repeat revascularization was significantly higher in the PCI group, and this was a persistent finding, regardless of adjustments, similar to findings reported in previous studies (13). A similar incidence of stroke has been reported after CABG or PCI in patients with CKD (13, 14, 18), although some studies have reported a lower risk of stroke after PCI (19). In this study, we found a higher incidence of stroke after CABG than PCI.

### Kidney-related events

Both PCI and CABG can cause kidney injury—PCI by contrast nephropathy (20), and CABG through renal hypoperfusion, inflammation, and decreased autoregulation (21). CKD is associated with a higher incidence of post-revascularization AKI (22, 23), but relatively few studies have compared the impacts of the two revascularization methods on renal function. Although several studies have found that AKI occurs more frequently after CABG than PCI, at least in the short-term period (5, 7), there is a paucity of data on long-term outcomes. Charytan et al. analyzed patients with CKD undergoing PCI or CABG and reported that rates of end-stage kidney disease were similar between the two groups (24). Although our definition of kidney-related secondary outcome differed from that of Charytan and colleagues, we observed a significantly higher incidence of renal outcomes in the CABG group than in the PCI group. The Kaplan–Meier curve demonstrated a large proportion of kidney-related events occurred during the early post-operation phase, while a landmark analysis showed that differences in the incidence rates between the groups were more prominent before than after the 30-day time point. Our results indicate that patients with CKD are at a relatively high risk of acute kidney-related events during the early post-CABG period, and close attention must be paid for optimal management after surgery. Landmark analysis showed a significant difference of incidence even after the 30-day time point, indicating that patients with CKD are at a long-term risk of kidney injury after CABG. Larger prospective studies with more sensitive measurements of kidney injury are needed to clarify the underlying cause(s).

### Difference according to completeness of revascularization

The benefits of PCI with CR compared with PCI with IR have been elucidated in various studies (8) but have not been consistently demonstrated in patients with CKD (25). We found a similar incidence of MACCE in the PCI-CR and CABG groups, while the PCI-IR group had worse outcomes. The risk of repeat revascularization was significantly lower after CABG than either type of PCI, implying that the risk of repeat revascularization stems from the characteristics of the PCI itself. PCI-CR was more protective on renal function than CABG based on Cox proportional hazard analysis, regardless of the adjustment method. Interestingly, the PCI-IR group did not have a significantly different incidence of renal composite outcomes from either the PCI-CR group or the CABG group. Due to this, it is unclear whether PCI-CR can really provide renal protection for patients with CKD compared to CABG or if this advantage is specific to our study setting. Moreover, the contrast volume used in the PCI-CR group was significantly larger than that used in the PCI-IR group, indicating a higher risk of contrast-induced nephropathy.

These findings altogether imply that PCI-CR can provide more favorable cardiovascular outcomes than PCI-IR can for coronary artery disease in CKD to a degree comparable to CABG and might also provide additional protection of kidney function. In practice, achieving CR is not always possible with PCI due to various factors, such as coronary anatomy and patients’ clinical status, so determining which method is better to achieve CR prior to treatment would be very important. In addition, it can be argued that if PCI is chosen as a revascularization method, CR must be attempted to warrant more favorable outcomes. Further studies are needed to evaluate the overall effects of complete revascularization on patients’ outcomes.

## Limitations

Drastic differences were found in baseline characteristics between the PCI and the CABG groups. PCI was more frequently performed in high-risk patients, such as those with old age, worse left ventricular systolic function, or diabetes mellitus. By contrast, patients with left main involvement and three-vessel disease were more likely to undergo CABG than PCI. To overcome this discrepancy, we established several exclusion criteria to balance the characteristics of the study population, adjusted for multiple factors, and performed matching by IPW analysis. However, these efforts had a limited effect, evidenced by relatively high standard mean differences even after weighted adjustment. Although our analysis implies that the CABG group is at higher risk of having kidney-related events, interpretation may be limited as we could not observe a definite trend of worsening GFRs over the follow-up period. A longitudinal curve regarding the GFR for each group may have provided the clearer message, but it was unavailable due to missing data and different follow-up periods between the two groups. In addition, we did not distinguish between CR and IR in the CABG group, possibly underestimating the effect of CR by CABG. Patients on dialysis were excluded because the renal composite outcome in this study included the first initiation of dialysis, thus limiting extrapolation of our findings to patients with end-stage renal disease. Finally, the creatinine value of the patients before the acute baseline event could not be identified in several cases, making it difficult to determine the presence of AKI at presentation, and the patients with AKI were not excluded from our study.

## Conclusion

The incidence of MACCE is similar after CABG and PCI, but kidney-related events are significantly more frequent after CABG, especially in the early post-procedure phase. Achieving CR with PCI might prevent the adverse outcome and improve the prognosis comparable to that of CABG with the possibility of more favorable outcomes in kidney function. Therefore, if PCI is chosen to treat coronary disease in patients with CKD, it is important that physicians try to achieve CR to ensure favorable outcomes regarding the patients’ cardiovascular and renal outcomes. Further large-scale studies are needed to confirm our findings.

## Data availability statement

The original contributions presented in this study are included in the article/Supplementary material, further inquiries can be directed to the corresponding author.

## Ethics statement

The studies involving human participants were reviewed and approved by the Institutional Review Board of Samsung Medical Center. Written informed consent for participation was not required for this study in accordance with the national legislation and the institutional requirements.

## Author contributions

KC and YS contributed to the conception and design of the study. WK and KC prepared the initial manuscript and performed the statistical analysis. SL organized the database. JL, TP, JY, J-YH, S-HC, SC, YC, KS, WSK, H-CG, and YL performed the procedure and registered the initial data to the database. All authors contributed to manuscript revision, and read and approved the submitted version.

## Abbreviations

CKD: chronic kidney disease
MI: myocardial infarction
CABG: coronary artery bypass grafting
PCI: percutaneous coronary intervention
AKI: acute kidney injury
CR: complete revascularization
IR: incomplete revascularization
GFR: glomerular filtration rate
IPW: inverse probability weighting.
