# Supplementary material for: Comparison of long-term cardiovascular and renal outcomes between percutaneous coronary intervention and coronary artery bypass grafting in multi-vessel disease with chronic kidney disease
Source: Front Cardiovasc Med. 2022 Sep 12;9:951113. doi: 10.3389/fcvm.2022.951113 (PMC9510652; doi:10.3389/fcvm.2022.951113)
Supplement: Supplementary file 1 [file Data_Sheet_1.docx]

**SUPPLEMENTAL MATERIALS**

**Comparison of long-term cardiovascular and renal outcomes between PCI and CABG in chronic kidney disease**

Woochan Kwon, MD^1✝^; Ki Hong Choi, MD, PhD^1^; Dong Seop Jeong, MD, PhD^2^; Sang Yoon Lee, MD^1^; Joo Myung Lee, MD, MPH, PhD^1^; Taek Kyu Park, MD, PhD^1^; Jeong Hoon Yang, MD, PhD^1^; Joo-Yong Hahn, MD, PhD^1^; Seung-Hyuk Choi, MD, PhD^1^; Su Ryeun Chung, MD^2^; Yang Hyun Cho, MD, PhD^2^; Kiick Sung, MD, PhD^2^; Wook Sung Kim, MD, PhD ^2^; Hyeon-Cheol Gwon, MD, PhD^1^; Young Tak Lee, MD, PhD^2✝^; Young Bin Song, MD, PhD^1*^

^1^Division of Cardiology, Department of Internal Medicine, Heart Vascular Stroke Institute, Samsung Medical Center, Sungkyunkwan University School of Medicine, Seoul, Republic of Korea

^2^Department of Thoracic and Cardiovascular Surgery, Heart Vascular Stroke Institute, Samsung Medical Center, Sungkyunkwan University School of Medicine, Seoul, Republic of Korea

**^✝^Dr. Kwon and Dr. Choi contributed equally to this work.**

**(1) Supplementary Method**

**(2) Supplementary Tables**

**(3) Supplementary Figures and Figure Legends**

**(4) References**

**(1) Supplementary Method**

**Procedures**

All coronary revascularization and related medical therapy were performed according to standard procedural guidelines.^1,2^ A loading dose of aspirin (300 mg) and P2Y12 inhibitor (clopidogrel 300-600 mg, ticagrelor 180 mg, or prasugrel 60 mg) before PCI was given unless the patients had been regularly taking the medications. Anticoagulation during PCI was performed using unfractionated heparin to achieve an activated clotting time of 250-300 seconds. Invasive coronary angiography was performed via a trans-radial or trans-femoral approach according to standard techniques. Diagnostic angiograms were obtained after intracoronary nitrate (100 or 200ug) administration. Percutaneous coronary intervention (PCI) was performed using current standard techniques with 2^nd^ generation drug-eluting stents or drug-eluting balloon. During PCI, type of stents or balloon, stenting techniques, use of additional imaging devices such as intravascular ultrasound or optical coherence tomography, or post-stent adjunctive balloon inflation were left to the operator’s discretion.

For CABG, Off-pump CABG utilizing the bilateral internal thoracic artery is the preferred technique for CABG at our institution.^3^ All operations were performed through standard median sternotomy. Bilateral internal thoracic arteries were prepared by skeletonization techniques with sharp dissection, clipping, and branch ligation. The saphenous vein was harvested from the patient's upper or lower leg with split incisions. Heparinized saline was used to dilate the diameter of the saphenous vein graft (SVG). The in situ right gastroepiploic artery (GEA) was prepared in a pedicled manner. The right internal thoracic artery (RITA) was anastomosed to the left side of the left internal thoracic artery (LITA) with a continuous running suture to construct a Y composite graft. With few exceptions, the LITA was anastomosed to the left anterior descending artery (LAD) and its branches, and then, the RITA was sequentially anastomosed to the left circumflex artery (LCX). If proximal right coronary artery (RCA) stenosis was greater than 80%, the RITA was initially selected as a graft. If the length of the harvested RITA was not sufficient to reach the RCA anastomosis, the right GEA was used. If proximal RCA stenosis was less than 80%, aortocoronary bypass was performed using the SVG. Perioperative treatment strategies including the use of cardiopulmonary bypass, number of grafts used, determination of anastomosis site, and concomitant medical therapy were left to the operator’s preference. A Transonic Flowmeter (Transonic Systems, Ithaca, NY, USA) was used to evaluate the quality of the anastomosis according to transit time flow rate.

**(2) Supplementary Tables**

**Supplementary Table 1. Standard mean deviations of 3 groups**

| **Variables** | **Unadjusted SMD** | | | **IPW-adjusted SMD** | | |
| --- | --- | --- | --- | --- | --- | --- |
|  | **CABG vs PCI-CR** | **CABG vs PCI-IR** | **PCI-CR vs PCI-IR** | **CABG vs PCI-CR** | **CABG vs PCI-IR** | **PCI-CR vs PCI-IR** |
| Age | 21.1 | 11.9 | -8.1 | 18.9 | 12.0 | -5.8 |
| Sex | 11.2 | -0.5 | -11.7 | 7.5 | -1.9 | -9.5 |
| Hypertension | 4.0 | -4.6 | -8.6 | 2.1 | -3.3 | -5.4 |
| Diabetes mellitus | 6.0 | 19.3 | 13.2 | 11.4 | 22.6 | 11.1 |
| History of myocardial infarction | -4.8 | 18.1 | 22.9 | -8.4 | 16.1 | 24.6 |
| History of PCI | -5.4 | 25.6 | 31.1 | -3.9 | 20.8 | 24.8 |
| History of CABG | 14.0 | 45.1 | 30.7 | 16.0 | 27.8 | 11.7 |
| History of stroke | -25.8 | -17.1 | 8.6 | -22.7 | -16.4 | 6.3 |
| Acute myocardial infarction at presentation | -7.9 | 16.5 | 24.5 | -1.9 | 15.8 | 17.7 |
| Pre-procedure LVEF | 21.6 | 15.3 | -7.4 | 13.5 | 14.0 | -0.2 |
| Creatinine | 1.6 | 5.5 | 3.3 | -1.3 | 9.3 | 9.9 |
| Hemoglobin | 30.3 | 20.4 | -9.9 | 19.0 | 7.5 | -11.5 |
| Left main involvement | -31.6 | -31.5 | 0.2 | -27.3 | -29.1 | -1.8 |
| 3-vessel disease | -93.8 | -55.5 | 33.9 | -32.8 | -31.4 | 1.3 |

SMD, standard mean deviation; IPW, inverse probability weighting; CABG, coronary artery bypass graft; PCI-CR, percutaneous coronary intervention with complete revascularization; PCI-IR, percutaneous coronary intervention with incomplete revascularization; LVEF, left ventricular ejection fraction; RAS, renin-angiotensin system

**Supplementary Table 2. Comparison of baseline characteristics among CABG, PCI-CR and PCI-IR**

| **Variables** | **CABG (N=443)** | **PCI** | | **P value**  **CABG vs PCI-CR** | **P value**  **CABG vs PCI-IR** | **P value**  **PCI-CR vs PCI-IR** |
| --- | --- | --- | --- | --- | --- | --- |
|  |  | **PCI-CR (N=132)** | **PCI-IR (N=223)** |  |  |  |
| **Demographics** |  |  |  |  |  |  |
| Age (years) | 70.0 [64.0, 74.0] | 71.0 [66.0, 75.0] | 71.0 [65.0, 75.0] | 0.02 | 0.03 | 0.66 |
| Male | 303 (68.4%) | 97 (73.5%) | 152 (68.2%) | 0.31 | 1.00 | 0.35 |
| Body mass index (kg/m^2^) | 24.6 [22.7, 26.8] | 24.5 [22.9, 25.9] | 24.8 [22.8, 26.8] | 0.39 | 0.45 | 0.17 |
| **Cardiovascular risk factors** |  |  |  |  |  |  |
| Hypertension | 352 (79.5%) | 107 (81.1%) | 173 (77.6%) | 0.78 | 0.65 | 0.52 |
| Diabetes mellitus | 293 (66.1%) | 91 (68.9%) | 167 (74.9%) | 0.62 | 0.03 | 0.28 |
| History of myocardial infarction | 43 (9.7%) | 11 (8.3%) | 35 (15.7%) | 0.76 | 0.03 | 0.07 |
| History of PCI | 83 (18.7%) | 22 (16.7%) | 66 (29.6%) | 0.68 | 0.002 | 0.01 |
| History of CABG | 7 (1.6%) | 5 (3.8%) | 26 (11.7%) | 0.23 | <0.001 | 0.02 |
| History of stroke | 83 (18.7%) | 13 (9.8%) | 28 (12.6%) | 0.02 | 0.06 | 0.55 |
| **Initial presentation** |  |  |  |  |  |  |
| Diagnosis at presentation |  |  |  |  |  |  |
| Acute myocardial infarction | 62 (14.0%) | 15 (11.4%) | 45 (20.2%) | 0.53 | 0.05 | 0.05 |
| Stable angina | 209 (47.2%) | 84 (63.6%) | 141 (63.2%) | 0.001 | <0.001 | 1.00 |
| Unstable angina | 172 (38.8%) | 33 (25.0%) | 37 (16.6%) | 0.01 | <0.001 | 0.07 |
| Pre-procedure LVEF (%) | 56.4 [42.5, 64.0] | 60.0 [47.0, 67.0] | 58.0 [46.0, 65.0] | 0.02 | 0.20 | 0.26 |
| LVEF < 40% at baseline | 105 (23.7%) | 45 (34.1%) | 80 (35.9%) | 0.02 | 0.001 | 0.82 |
| Creatinine (mg/dL) | 1.4 [1.2, 1.7] | 1.3 [1.2, 1.6] | 1.4 [1.3, 1.7] | 0.56 | 0.70 | 0.36 |
| Glomerular filtration rate (mL/min/1.73m^2^) | 49.5 [38.8, 55.9] | 52.0 [39.6, 55.8] | 50.2 [36.7, 55.7] | 0.39 | 0.71 | 0.31 |
| Hemoglobin (mg/dL) | 11.9 [10.5, 13.3] | 12.1 [11.0, 14.0] | 12.0 [11.0, 14.0] | 0.002 | 0.01 | 0.39 |
| C-reactive protein (mg/L) | 0.2 [0.1, 0.8] | 0.1 [0.0, 0.3] | 0.2 [0.1, 0.6] | 0.001 | 0.32 | 0.02 |
| **Coronary anatomy** |  |  |  |  |  |  |
| Left main involvement | 79 (17.8%) | 10 (7.6%) | 17 (7.6%) | 0.01 | 0.001 | 1.00 |
| Number of involved vessels |  |  |  | <0.001 | <0.001 | 0.003 |
| 2-vessel disease | 93 (21.0%) | 83 (62.9%) | 103 (46.2%) |  |  |  |
| 3-vessel disease | 350 (79.0%) | 49 (37.1%) | 120 (53.8%) |  |  |  |
| **Medication at the baseline** |  |  |  |  |  |  |
| Aspirin | 430 (97.1%) | 132 (100.0%) | 221 (99.1%) | 0.10 | 0.16 | 0.72 |
| P2Y12 inhibitor | 322 (72.7%) | 131 (99.2%) | 222 (99.6%) | <0.001 | <0.001 | 1.00 |
| Beta blocker | 372 (84.0%) | 81 (61.4%) | 139 (62.3%) | <0.001 | <0.001 | 0.95 |
| RAS blocker | 121 (27.3%) | 102 (77.3%) | 162 (72.6%) | <0.001 | <0.001 | 0.40 |
| Statin | 317 (71.6%) | 121 (91.7%) | 209 (93.7%) | <0.001 | <0.001 | 0.61 |

Values are mean ± standard deviation (or median [1^st^ interquartile, 3^rd^ interquartile]) or number (%).

CABG, coronary artery bypass graft; CABG, coronary artery bypass graft; PCI-CR, percutaneous coronary intervention with complete revascularization; PCI-IR, percutaneous coronary intervention with incomplete revascularization; LVEF, left ventricular ejection fraction; RAS, renin-angiotensin system

**Supplementary Table 3. Baseline lesion and procedural characteristics of PCI group**

| **Variables** | **All PCI (N=355)** | **PCI-CR (N=132)** | **PCI-IR (N=223)** | **P-value*** |
| --- | --- | --- | --- | --- |
| **Lesion characteristics** | | | | |
| Lesion location (per vessel) | | | | |
| Left main coronary artery | 27 (7.6%) | 10 (7.6%) | 17 (7.6%) | 0.99 |
| Left anterior descending artery | 315 (88.7%) | 118 (89.4%) | 197 (88.3%) | 0.76 |
| Left circumflex artery | 280 (78.9%) | 99 (75.0%) | 181 (81.2%) | 0.17 |
| Right coronary artery | 269 (75.8%) | 91 (68.9%) | 178 (79.8%) | 0.02 |
| Number of lesion (per vessel) | 3.0 [2.0, 4.0] | 3.0 [2.5, 4.0] | 3.0 [2.0, 4.0] | 0.53 |
| Chronic total occlusion | 108 (30.4%) | 26 (19.7%) | 82 (36.8%) | 0.001 |
| In-stent restenosis | 22 (6.2%) | 8 (6.1%) | 14 (6.3%) | 1.00 |
| Bifurcation lesion | 84 (23.7%) | 40 (30.3%) | 44 (19.7%) | 0.03 |
| Mean pre-PCI diameter stenosis (%) | 86.3 [76.7, 92.5] | 82.5 [80.0, 89.0] | 90.0 [75.0, 95.0] | 0.002 |
| Mean lesion length (mm) | 10.0 [6.0, 15.7] | 15.2 [11.0, 21.0] | 7.5 [4.4, 11.0] | <0.001 |
| SYNTAX score | 15.0 [10.0, 23.8] | 15.0 [10.0, 24.5] | 15.0 [9.0, 23.0] | 0.54 |
| **Procedure characteristics** | | | | |
| Transradial approach | 265 (74.6%) | 107 (81.1%) | 158 (70.9%) | 0.04 |
| Contrast volume (ml) | 200.0 [150.0, 250.0] | 230.0 [182.5, 272.5] | 180.0 [140.0, 220.0] | 0.003 |
| Fluoroscopy time (minutes) | 18.5 [12.0, 26.4] | 21.2 [14.5, 27.4] | 17.3 [10.4, 25.7] | 0.003 |
| Implanted stent number | 2.0 [1.0, 2.0] | 3.0 [2.0, 4.0] | 1.0 [1.0, 1.0] | <0.001 |
| Mean stent diameter (mm) | 3.0 [2.7, 3.1] | 2.9 [2.7, 3.1] | 3.0 [2.8, 3.1] | 0.62 |
| Mean stent length (mm) | 24.0 [20.0, 28.0] | 24.7 [22.0, 28.0] | 24.0 [18.8, 28.0] | 0.06 |
| Use of intravascular imaging | 70 (19.7%) | 31 (23.5%) | 39 (17.5%) | 0.22 |
| Use of Rotablation | 12 (3.4%) | 4 (3.1%) | 8 (3.6%) | 0.78 |
| Drug eluting balloon | 9 (2.5%) | 4 (3.1%) | 5 (2.2%) | 0.43 |
| Used stent types |  |  |  | 0.02 |
| Durable polymer, everolimus-eluting stent | 128 (36.1%) | 54 (41.0%) | 74 (33.2%) |  |
| Biodegradable polymer, everolimus-eluting stent | 66 (18.6%) | 23 (17.3%) | 43 (19.3%) |  |
| Zotarolimus-eluting stent | 72 (20.3%) | 21 (15.9%) | 51 (22.8%) |  |
| Biolimus coated stent | 37 (10.4%) | 13 (9.9%) | 24 (10.7%) |  |
| Others | 43 (12.1%) | 17 (12.9%) | 26 (11.7%) |  |

^*^P-value for comparison between the PCI-CR group and the PCI-IR group

Values are mean ± standard deviation (or median [1^st^ interquartile, 3^rd^ interquartile]) or number (%).

PCI, percutaneous coronary intervention; PCI-CR, percutaneous coronary intervention with complete revascularization; PCI-IR, percutaneous coronary intervention with incomplete revascularization

**Supplementary Table 4. Baseline procedural characteristics of CABG group**

| **Variables** | **Number** |
| --- | --- |
| Off-pump CABG | 350 (79.0%) |
| Number of anastomoses | 4.0 [3.0, 5.0]^*^ |
| Use of left internal mammary artery | 431 (97.3%) |
| Use of right internal mammary artery | 402 (90.7%) |
| Use of bilateral mammary artery | 394 (88.9%) |
| Use of other arterial grafts^†^ | 29 (6.5%) |
| Use of saphenous venous graft | 109 (24.6%) |

^*^Value is presented as median [1^st^ interquartile, 3^rd^ interquartile].

^†^Other arterial grafts included radial artery and right gastroepiploic artery

CABG, coronary artery bypass graft

**(3) Supplementary Figures and Figure Legends**

**
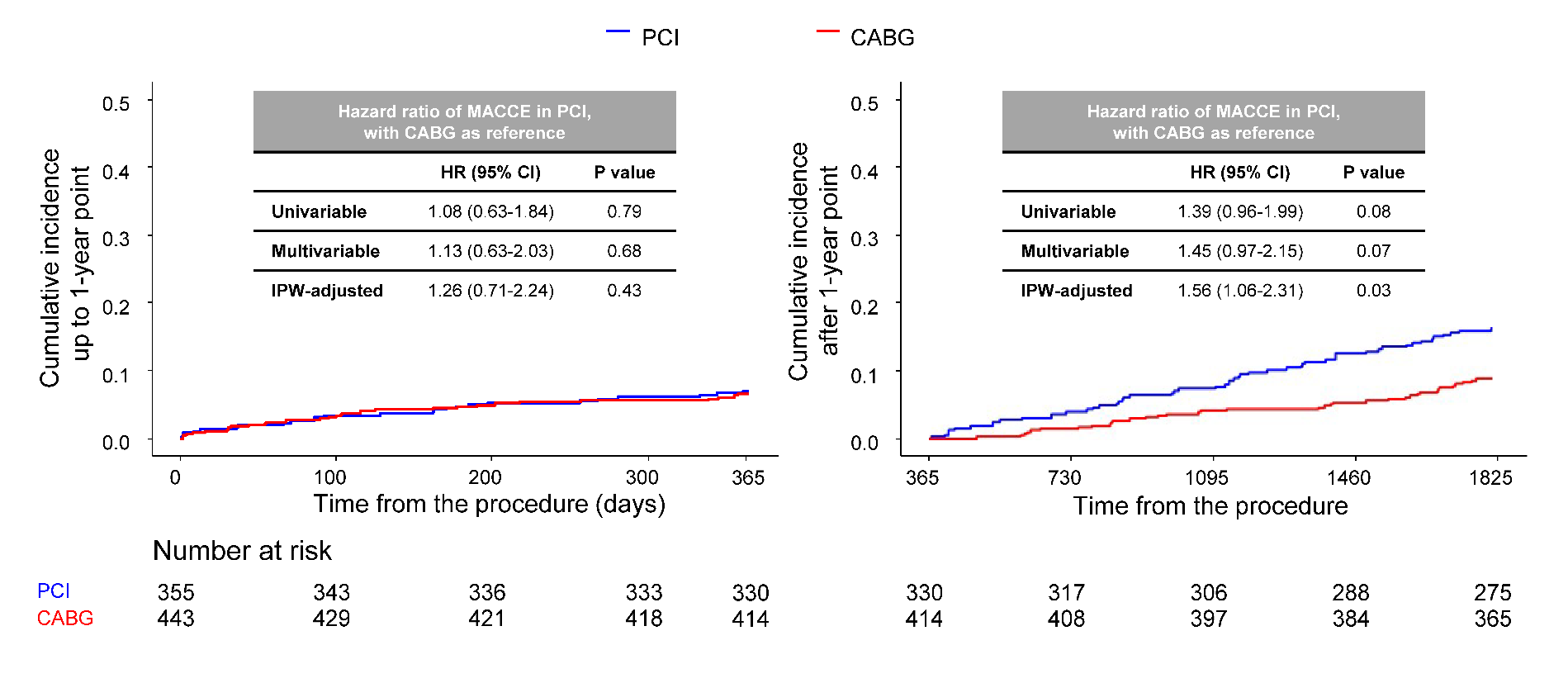
**

**Supplemental Figure 1. Landmark analysis at the time point of 1-year between CABG and PCI.**

Kaplan-Meier curve comparing 5-year MACCE between the PCI and CABG groups with landmark analysis before **(left)** and after **(right)** 1 year.

CABG, coronary artery bypass graft; PCI, percutaneous coronary intervention; MACCE, major cardiac and cerebrovascular events

**
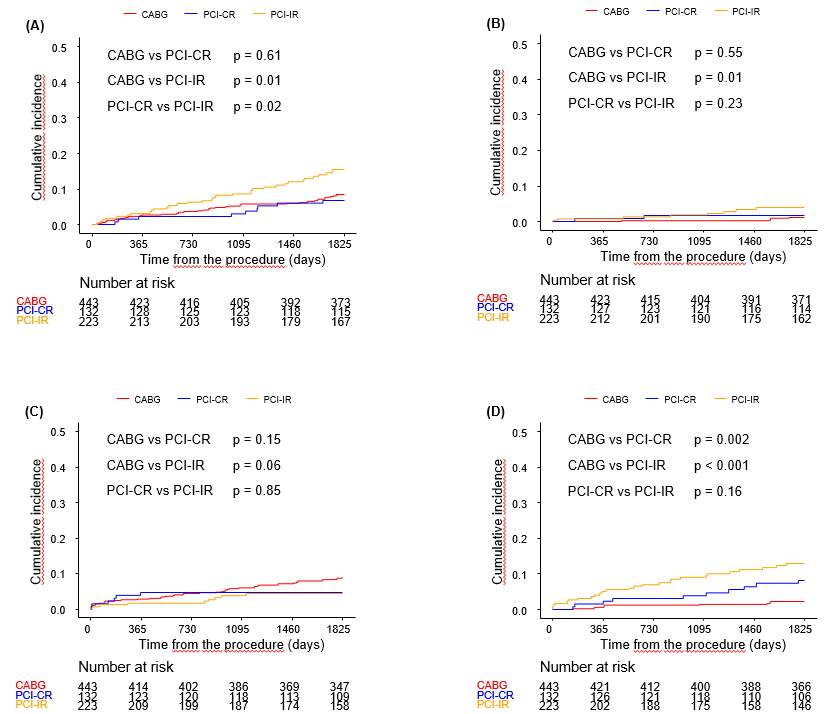
**

**Supplementary figure 2. Kaplan-Meier curve comparing (A) cardiac death (B) acute myocardial infarction (C) stroke (D) repeat revascularization at 5-year point among the CABG and two PCI groups** CABG, coronary artery bypass graft; PCI, percutaneous coronary intervention; PCI-CR, percutaneous coronary intervention with complete revascularization; PCI-IR, percutaneous coronary intervention with incomplete revascularization

**(4) References**

1. Neumann FJ, Sousa-Uva M, Ahlsson A, Alfonso F, Banning AP, Benedetto U, Byrne RA, Collet JP, Falk V, Head SJ, et al. 2018 ESC/EACTS Guidelines on myocardial revascularization. *EuroIntervention*. 2019;14:1435-1534. doi: 10.4244/EIJY19M01_01

2. Lawton JS, Tamis-Holland JE, Bangalore S, Bates ER, Beckie TM, Bischoff JM, Bittl JA, Cohen MG, DiMaio JM, Don CW, et al. 2021 ACC/AHA/SCAI Guideline for Coronary Artery Revascularization: Executive Summary: A Report of the American College of Cardiology/American Heart Association Joint Committee on Clinical Practice Guidelines. *Journal of the American College of Cardiology*. 2021. doi: 10.1016/j.jacc.2021.09.005

3. Choi KH, Song YB, Jeong DS, Jang YH, Hong D, Lee SY, Youn T, Bak M, Min KM, Lee JM, et al. Differential effects of dual antiplatelet therapy in patients presented with acute coronary syndrome vs. stable ischaemic heart disease after coronary artery bypass grafting. *Eur Heart J Cardiovasc Pharmacother*. 2021;7:517-526. doi: 10.1093/ehjcvp/pvaa080
