# Supplementary figures and images for: Comparison of long-term cardiovascular and renal outcomes between percutaneous coronary intervention and coronary artery bypass grafting in multi-vessel disease with chronic kidney disease
Source: Front Cardiovasc Med. 2022 Sep 12;9:951113. doi: 10.3389/fcvm.2022.951113 (PMC9510652; doi:10.3389/fcvm.2022.951113)

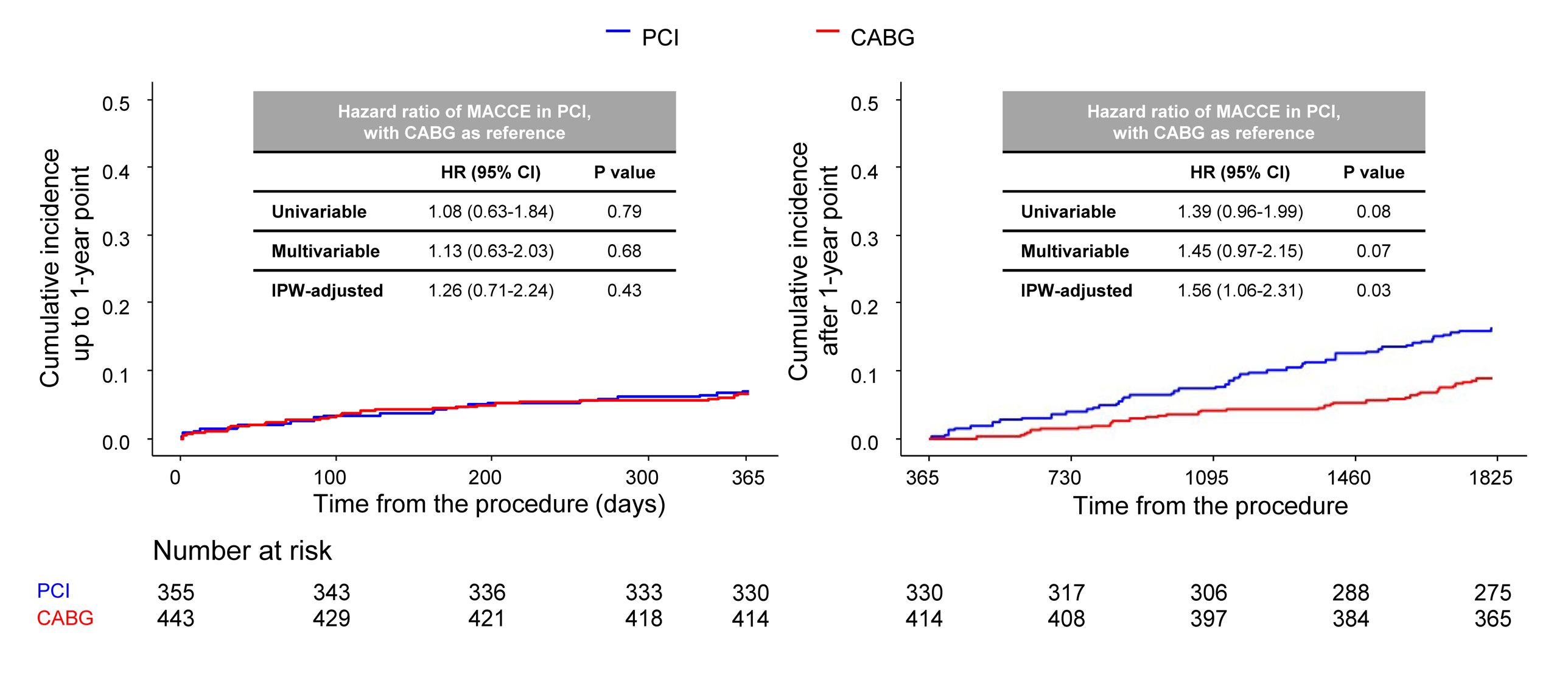

Supplement: Supplementary file 2 [file Image_1.TIF]
